# Editorial: Women in neuropharmacology 2023

**DOI:** 10.3389/fphar.2024.1441780

**Published:** 2024-07-31

**Authors:** Paola Sacchetti

**Affiliations:** Department of Biology, University of Hartford, West Hartford, CT, United States

**Keywords:** gender equality, neuropsychiatry, substance abuse, ADHD (attention deficit and hyperactivity disorder), cocaine, alcohol, tobacco, CBD-cannabidiol

## Introduction

The field of neuropharmacology delves into the intricate relationship between drugs and the nervous system. By investigating how these drugs impact brain function, researchers gain insights into normal neural processes and discover potential avenues for treating neurological and psychiatric disorders. These disorders include depression, anxiety, substance abuse, Alzheimer’s disease, and Parkinson’s disease.

Despite being underrepresented, women have significantly shaped the advancement of neuropharmacology and our understanding of the brain. Notable female neuroscientists have made substantial contributions, particularly in the realm of substance abuse research. Exceptional figures, such as Dr. Mary Jeanne Kreek, a pivotal figure in understanding heroin addiction as a neurological disorder and in identifying methadone as an effective treatment for withdrawal symptoms (Dole et al., 1966), and Dr. Candace Pert, whose groundbreaking discoveries include identifying the opiate receptor in the brain (Pert and Snyder, 1973) and advancing our understanding of neuropeptides, lead the way. Others, like Dr. Yasmin Hurd, who has conducted groundbreaking research on cannabis and its effects during prenatal exposure (Hurd et al., 2019), are shedding light on critical areas with societal implications. Although too slowly, excellent female scientists are reaching leadership positions, such as Dr. Nora Volkow, a brain imaging pioneer in drug addiction research, who currently serves as the director of the National Institute on Drug Abuse (NIDA) (National Institute on Drug Abuse, 2024). These remarkable women have not only advanced various subfields within neuropharmacology but have also underscored the vital role women scientists play in shaping decision-making processes. Their contributions pave the way for new treatments and inspire future generations of researchers.

The current collection aims to elevate the contributions of women scientists across all fields of neuropharmacology. Comprising four articles, this Research Topic primarily focuses on cutting-edge research related to substance abuse. Notably, two of the selected articles feature women as first authors, and two originate from laboratories led by women. Overall, these publications boast predominantly female authorship.

Castelli et al. explore the potentials of cannabidiol (CBD) in reversing hippocampal deficiencies in rats prenatally exposed to tetrahydrocannabinol (pTHC). Their results support the knowledge that prenatal THC exposure impairs hippocampal memory and synaptic plasticity in adolescent male Wistar rats. Remarkably, CBD injections during adolescence can mitigate spatial memory deficits in pTHC-exposed rats and reduce overexpression of excitatory synapse markers in the hippocampus. These results open avenues to further investigating CBD as a potential rescue strategy in counteracting cannabis-related effects.

Lori Knackstedt’s group evaluated the effects of sequential cocaine and alcohol polysubstance use (PSU) *versus* cocaine alone on activation of reward circuitry brain areas in male Sprague Dawley rats Mesa et la. They established that only the prelimbic cortex showed differential effects due to alcohol consumption and Ceftriaxone, an antibiotic known to attenuate reinstatement of cocaine-seeking behavior, did not alter activation in PSU-induced conditions. Understanding the distinct reward neurocircuitry activation patterns between PSU and single cocaine use is vital to identifying effective therapies against drug relapse.

Vicarelli et al. investigate the impact of unburned tobacco smoke on the prefrontal cortex (PFC) in male Sprague Dawley rats. Their findings shed light on the intricate interplay between oxidative stress, inflammation, and neurotoxicity. Their results show that exposure to neurotoxic and carcinogenic compounds derived from “heat-not-burn” tobacco increases radical species that could give rise to the DNA damage, oxidative stress and neuroinflammation observed in the PFC. Vicarelli et al. research underscores the need for vigilance regarding unburned tobacco smoke and its potential consequences on brain health.

Sader Nehme et al. study the role of the ATP receptor P2X4 in the context of Attention Deficit Hyperactivity Disorder (ADHD). They demonstrate that the complete absence of the P2X4 receptor (P2X4KO) prevents locomotor hyperactivity in the well-established 6-hydroxydopamine lesion model of ADHD but does not fundamentally alter pain sensitivity in these mice. Interestingly, the absence of P2X4 induces changes in microglia reactivity and neuroinflammation in a region-specific manner. Understanding how purinergic signaling influences neuroinflammation could provide new avenues to identify ADHD-specific therapeutic interventions.

## Conclusion

The current collection highlights the valuable contributions of women researchers in the field of neuropharmacology. These accomplished scientists lead important investigations into neurological disorders and substance abuse, significantly impacting our understanding of brain function.

Whether serving as first or last authors, women scientists play pivotal roles in advancing our knowledge of brain functionality and disorders. Through their leadership in research activities, they make significant contributions to the field’s progress. In addition to their individual achievements, women scientists excel as team members, collaborating to make important discoveries that benefit diverse research groups. Their ability to work effectively within teams enhances the collective scientific endeavor. It is promising that roughly 50% of trainees participating in the Society for Neuroscience meetings are women, but it is concerning that only 30% of the faculty attendees are women. Furthermore, in neuroscience journals analyzed between 2009–2018, while women were the first author in 49% of published manuscripts, they were the last author in only 31% of them (Schrouff et al., 2019). Additionally, women were cited in high-impact factor biomedical journals at much lower rates than men (16% vs. 84%) between 2014–2020 (Shamsi et al., 2022). Authorship and citations can significantly impact grant applications and promotion procedures, especially for junior researchers. Therefore, it is crucial to provide high-quality mentorship and resources to support women in the laboratory. However, the need for support in developing robust publishing track records and creating a supportive and inclusive publishing system for all researchers seems of the essence. Empowering women scientists at all levels of their careers will enrich all fields of science, and society as a whole, and we must all be prepared to take proactive steps to enhance gender equality.
